# A framework for implementation of community-orientated primary care in the Metro Health Services, Cape Town, South Africa

**DOI:** 10.4102/phcfm.v12i1.2632

**Published:** 2020-12-18

**Authors:** Robert Mash, Charlyn Goliath, Hassan Mahomed, Steve Reid, Derek Hellenberg, Gio Perez

**Affiliations:** 1 Division of Family Medicine and Primary Care, Faculty of Medicine and Health Sciences, Stellenbosch University, Cape Town, South Africa; 2 Metro Health Services, Western Cape Government, Cape Town, South Africa; 3 Department of Global Health, Faculty of Medicine and Health Sciences, Stellenbosch University, Cape Town, South Africa; 4 Primary Health Care Directorate, University of Cape Town, Cape Town, South Africa; 5 Division of Family Medicine, University of Cape Town, Cape Town, South Africa

**Keywords:** primary health care, community-orientated primary care, primary care, service delivery, health system design, community health workers

## Abstract

In South Africa, the national policy on re-engineering primary health care (PHC) supports the implementation of ward-based outreach teams with community health workers. In the Western Cape, a community-orientated primary care (COPC) approach has been adopted in provincial goals for 2030 and the key strategies for the improvement of district health services. This approach is expected to improve health and also save costs. A task team was established in the Metropolitan Health Services to develop an implementation framework for COPC. The framework was developed in an iterative process with four learning sites in the metropole over a period of 18 months. The framework consists of 10 inter-related elements: geographic delineation of PHC teams, composition of PHC teams, facility-based and community-based teamwork, partnership of government and non-government organisations, scope of practice, information system, community engagement, stakeholder engagement, training and development of PHC teams, system preparation and change management. This framework was implemented at the four learning sites and is now being taken to scale and further assessed in the metropole.

## Introduction

The National Department of Health in South Africa included ward-based outreach teams as one of the key strategies to re-engineer primary health care (PHC).^1^ Ward-based outreach teams were conceptualised as teams of community health workers (CHW), led by a nurse and facilitating outreach into the community from a local primary care facility.^2^

The Western Cape Government in their ‘Healthcare 2030’ policy emphasised the need to adopt a person-centred approach across the whole lifecycle, which strengthened health promotion, disease prevention and PHC.^3^ Furthermore, the district health services (DHS) strategy document stated that:

Healthcare 2030 directs the Province to implement a population orientated community based DHS platform built on teams linked to PHC facilities which provide comprehensive outreach care based on a nurse driven service. The community orientated primary care (COPC) approach based on asset based thinking, utilising existing good practice models will inform the development of this aspect of the reform process.^4^

The adoption of a COPC approach was driven by the need to address a large quadruple burden of disease in the context of increasing budget constraints.^5^ The South African Medical Research Council modelled the implementation of a CHW platform, and despite a conservative approach, all interventions by CHWs in the fields of mother and child health, HIV/AIDs, TB, hypertension and diabetes would lead to a decrease of just under 200 000 deaths over 10 years and a saving of 4.8 million disability-adjusted life years.^6^ The return on investment was seen as very favourable with a projected saving of R2.4 billion over 10 years. CHWs therefore could improve health status and save costs.

Internationally, there is a body of evidence supporting the effectiveness of COPC in other regions, such as in Brazil, the United States, Turkey, Cuba and Spain.^7,8,9,10^ Most recent studies on COPC in Africa have focused on the implementation and not effectiveness, although there is recent evidence from Kenya^11^, and historically the concept of COPC was developed in South Africa and shown to be effective.^12^

The Metropolitan Health Services (MHS) component of the Western Cape Government: Health provincial health service established a task team to create a framework for the re-organisation of PHC according to a COPC approach. The MHS adopted the definition of COPC as:

[*A*] continuous process by which primary health care is provided to a defined community on the basis of its assessed health needs, by the planned integration of primary care practice and public health.^13^

## Task team process

The MHS is divided into four sub-structure areas to serve the population of Cape Town. The task team visited each sub-structure to identify existing examples of COPC and to share the new vision with local managers. Four learning sites (Mamre, Nomzamo, Eastridge and Bishop Lavis) were selected, where the emerging COPC framework could be both developed and implemented in an iterative process with the key stakeholders. Key stakeholders included the leadership of the local primary care facility and non-profit organisation (NPO), who employed the CHWs. In addition sub-structure management included at least the director, primary care manager (overseeing the primary care facilities) and comprehensive health manager (overseeing the NPOs).

A draft framework was developed based on the literature,^2,7,8,9,10,13^ health policy directions^3,4^ and initial visits to each sub-structure. The task team then held a series of workshops every 2–3 months with the stakeholders from each substructure over a period of 18 months (2017–2018) to present, develop and finalise the framework. Workshops were facilitated by the task team members. These workshops also helped stakeholders to share their initial steps to implement the framework and reflect on the appropriateness of the framework.

The final framework was revised by the task team, presented to the management of the Metro Health Services and formally adopted in 2019.

## The framework

**FIGURE 1:**
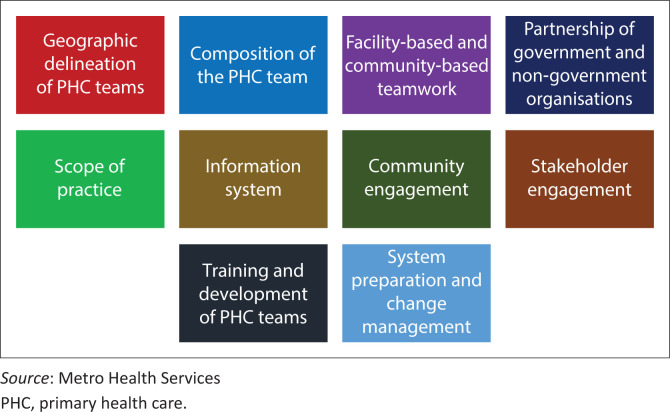
The 10 elements of the community-orientated primary care framework.

The framework comprised 10 inter-connected elements to guide the implementation of COPC (Figure 1).

### Geographic delineation of primary health care teams

**FIGURE 2:**
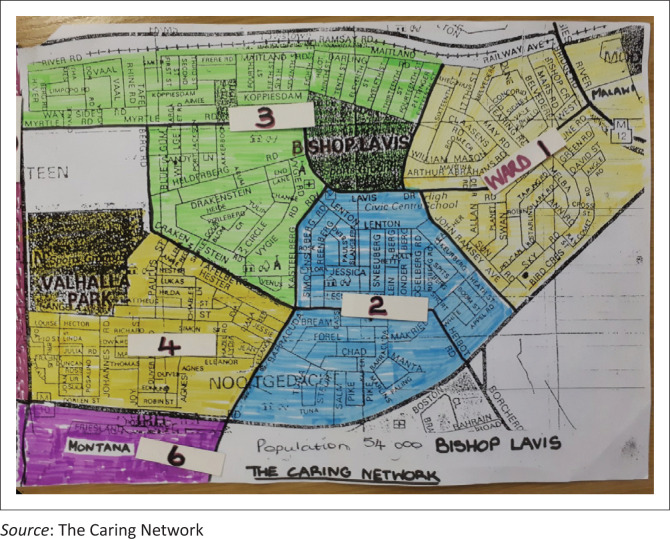
Delineation of Bishop Lavis community into PHC team areas.

The catchment areas of primary care facilities should be defined geographically in a contiguous manner through the sub-district so that the whole population is covered (Figure 2). Within each facility’s catchment area a number of sub-places (suburbs/township areas) are identified. Each sub-place is served by a PHC team. The number of teams required is therefore defined by the number of sub-places. As far as the PHC teams are concerned the NPOs should also operate in a geographically contiguous manner and without geographic overlap between NPOs.

### Composition of the primary health care team

The ideal composition of the PHC team to serve a sub-place was defined as:

- 10–15 CHWs (where 1 CHW was responsible for 250 households or approximately 1000 people).
- One team leader from within the CHW team.
- One professional nurse, one clinical nurse practitioner and one medical officer, all of whom may be responsible for or connected to several CHW teams.

The professional nurse and CHWs were employed by the NPO, whilst the nurse practitioner and medical officer were employed at the primary care facility. It was acknowledged that the final composition of each PHC team would depend on the local context and variables such as CHW working hours, population density, the community’s socio-economic status and burden of disease, distance to a primary care facility and staff mix at the facility.

A number of other professionals would be needed to support the functioning of the PHC team as required. These included the family physician, dietician, physiotherapist, occupational therapist, pharmacist, hospital-based specialists or social worker. In some areas, the team might also include a community-based rehabilitation worker.

The resources required (e.g. uniforms, equipment and stationary), travel and administrative costs for proper functioning of the PHC teams must also be defined and provided for.

### Facility-based and community-based teamwork

The PHC team members, whether employed through the NPO or at the facility, need to build a relationship and function as an integrated team. This would require regular meetings to build relationships, coordinate activities, plan training, discuss problems or challenges and review information of the health needs of the community.

The integration of PHC teams will require leadership from sub-structure, facility and NPO managers as well as the local family physician. The relationship between the professional nurse in the CHW team and the facility manager will be particularly critical. There may be some infrastructural requirements at the facility to support teamwork such as space for CHWs to meet or for the professional nurse.

### Partnerships of Western Cape Government with non-profit organisations

The Western Cape has adopted a model of employing CHWs through local NPOs rather than directly. The NPOs receive funding, deliver and report on a package of services according to a contract with the DOH which is currently managed by the comprehensive service manager in the sub-structure. An adapted contract was needed in order to align with the framework for COPC as, for example, NPOs sometimes overlapped geographic boundaries or focused on a specific disease or type of service. The contract should also be revised to ensure that the terms and conditions of employment provide sufficient capacity to deliver the new framework and are aligned with the relevant legislation (e.g. *Amended Labour Relations Act*). CHWs need to be paid a living wage and ideally work a full day so that they are more committed to the work in the long term. The managerial oversight of the PHC team should be integrated at the level of the sub-structure to avoid fragmentation of supervision and accountability.

### Scope of practice

The scope of practice needed to be re-defined for all members of the PHC team.

The scope of practice of CHWs must be a generalist one that includes the whole lifecycle (all age groups) and is comprehensive (appropriate health promotion, disease prevention, care and treatment, rehabilitative and palliative care across the burden of disease). The scope of practice needs to be feasible in terms of the CHWs’ capability, resources and time.

Practice at the household level implies that all households within a given sub-place should be registered with a PHC team. The level of health risk found amongst the members of each household should be determined as either no risk, low-risk, high-risk or an emergency, in order to plan further intervention or support. The need for health promotion, disease prevention or home-based care will be determined. PHC teams will refer clients appropriately to other services and may receive referrals for follow-up of clients in the community.

PHC teams will also practice in the community and contribute to other interventions such as support groups, wellness centres, workplace health promotion, health campaigns, school-based or early childhood development centre-based activities. PHC teams will also contribute to community engagement, diagnosis and prioritisation of health needs as well as planning of interventions.

Professional nurses in the CHW team should not only monitor CHW performance but coordinate care with the facility, provide in-service training of CHWs and clinical care for clients in support of CHWs where needed.

The nurse practitioner, medical officer and family physician at the facility must also support the CHW team with clinical decision making, training and feedback on referrals.

Targets should be generalist orientated and driven by household needs, not by external vertical disease-orientated programmes. Details of scope of practice may need to be adjusted to the specific needs of the local community.

### Information system

There should be a process of defining and clarifying data and information needs. For efficiency reasons, CHW teams should have a mobile electronic device for collecting data from households and an information system that collates and analyses data not only to supervise performance but also to provide information on the health needs of the community. This will contribute to a community diagnosis, prioritisation and intervention process. The system should also provide CHWs with records of visits at the household level to support continuity of information for follow up by the PHC team. The system should integrate with the rest of the DHS IT system with the potential to link data collected at the household level with data collected at the facility level on individual patients. The system should also provide the necessary data for the selected indicators at district, provincial and national levels. The PHC teams, sub-structure managers and district management team should also have access to the information system to extract reports at different levels.

### Community engagement

Engagement of health service staff with the communities that they serve happens formally through clinic committees, health forums (multiple clinic committees) and hospital boards. Municipal wards may also have health committees. At a higher level, District Health Councils are supposed to provide a place for formal engagement. However, these structures do not allow for the participation of more ordinary community members and other important stakeholders, and additional mechanisms need to be found to engage the whole community on health matters of importance.

A forum is needed that is open to all relevant stakeholders (from community, governmental, non-governmental and private sectors) within the geographic area served by the facility and its PHC teams. Such a forum will enable engagement with the community to co-define health resources available, health needs and priorities.

### Stakeholder engagement

Stakeholder engagement has the following goals:

- To collaborate with other health service providers to implement the COPC framework, particularly the City of Cape Town Municipality as a partner PHC provider.
- To engage with other stakeholders that can contribute to the work of the PHC teams or provide services to people referred by CHWs: private practitioners; alternative, complementary and traditional practitioners; other NPOs or governmental organisations (e.g. department of social development, education). Engagement with social services is particularly important.
- To engage with other stakeholders that can help address the social or environmental determinants of health such as NPOs, environmental health officers, education, urban planning.
- To foster a spirit of collaboration in addressing the development needs of deprived communities.

Networking, building relationships and collaboration needs to happen at many levels from the local PHC team, the NPO and primary care facility to the sub-structure and the MHS.

### Training and development of the primary health care teams

The scope of practice, learning outcomes and curriculum for the pre-service training of the PHC team, and especially the CHWs, needs to be agreed on and co-ordinated at the provincial level and with educational institutions. A standardised approach to the training of new CHWs should be developed by the DOH’s People’s Development Centre. New CHWs should have a matric qualification for entry to the job. Training should also enable CHWs to acquire formal National Qualification Framework (NQF) credits at the appropriate level. Attention should be given to career pathways for CHWs.

This initial training should be followed by ongoing weekly in-service training and incremental capacity building in the CHW team that is co-ordinated by the NPO. Training of CHWs should not only rely on formal classroom-based teaching but involve workplace-based adult experiential learning. Capability is gained by learning that is directed towards the specific health needs, problems and challenges which arise from the work. PHC team members should contribute to the training sessions.

Clinical staff at the facility need to be re-orientated and upskilled for a COPC approach, including training and mentoring CHWs, analysing and interpreting information from the CHW teams and community-level activities. Appropriate postgraduate courses and diplomas that support the development of the PHC team should be identified and made available.

The supervisory staff, such as the CHW team leader and professional nurse, will also need specific training for their roles.

### System preparation and change management

A strategy is needed to ensure that all clinical, support and managerial staff within the DOH are aware of and share a similar understanding of the new COPC framework. In addition, there is a need to inform communities about the new COPC framework and to elicit their support for the PHC teams working in their communities.

There are multiple initiatives at national and provincial levels to reform the health system overall with a special focus on the PHC level. These initiatives provide a policy and legislative framework within which this intervention operates. There needs to be ongoing communication with management structures at all levels on the implementation of COPC to ensure ongoing support. Key challenges with working conditions, changes in policy, budget implications and relationships with external stakeholders need to be identified and managed formally through available mechanisms. Once decisions have been reached, these should be clearly communicated, consulted and effectively implemented in partnership with affected persons as part of the change process.

## Conclusion

This short report outlines the rationale for the development of a framework for COPC in Cape Town and describes the 10 inter-related elements of the framework. The framework has been used to implement COPC in four learning sites and thereafter throughout the MHS. Further evaluation of the implementation of COPC in the MHS is underway and will be reported on in future.
